# Computer Vision for Parkinson’s Disease Evaluation: A Survey on Finger Tapping

**DOI:** 10.3390/healthcare12040439

**Published:** 2024-02-08

**Authors:** Javier Amo-Salas, Alicia Olivares-Gil, Álvaro García-Bustillo, David García-García, Álvar Arnaiz-González, Esther Cubo

**Affiliations:** 1 Escuela Politécnica Superior, Departamento de Ingeniería Informática, Universidad de Burgos, 09001 Burgos, Spaindgarcia1@ubu.es (D.G.-G.); 2 Facultad de Ciencias de la Salud, Departamento de Ciencias de la Salud, Universidad de Burgos, 09001 Burgos, Spain; agbustillo@ubu.es; 3 Servicio de Neurología, Hospital Universitario de Burgos, 09006 Burgos, Spain

**Keywords:** Parkinson’s disease, finger tapping, machine learning, computer vision

## Abstract

Parkinson’s disease (PD) is a progressive neurodegenerative disorder whose prevalence has steadily been rising over the years. Specialist neurologists across the world assess and diagnose patients with PD, although the diagnostic process is time-consuming and various symptoms take years to appear, which means that the diagnosis is prone to human error. The partial automatization of PD assessment and diagnosis through computational processes has therefore been considered for some time. One well-known tool for PD assessment is finger tapping (FT), which can now be assessed through computer vision (CV). Artificial intelligence and related advances over recent decades, more specifically in the area of CV, have made it possible to develop computer systems that can help specialists assess and diagnose PD. The aim of this study is to review some advances related to CV techniques and FT so as to offer insight into future research lines that technological advances are now opening up.

## 1. Introduction

In 2015, the Global Burden of Disease study estimated that neurological disorders are the leading cause of disability worldwide. The incidence and prevalence of neurodegenerative diseases such as Parkinson’s disease (PD) increase considerably with age. PD is the second most common neurodegenerative disease worldwide. According to the literature, the number of cases estimated between 1990 and 2015 doubled, affecting 6.2 million people worldwide, a figure that is likely to double again by 2040 [1].

PD is characterized by progressive primary motor disabilities following the degeneration of the dopaminergic neurons located in the substantia nigra and associated areas of the brain [2]. The pathophysiology of PD involves signature abnormalities in several parallels and largely segregated basal ganglia thalamocortical circuits (i.e., the motor circuit). The available evidence suggests that the varied movement disorders resulting from dysfunctions within that circuit result from the propagated disruption of downstream network activity in the thalamus, cortex, and brainstem, and neurotransmitters, including dopamine, acetylcholine, noradrenaline, and serotonin [3,4].

Although PD is mainly associated with motor symptoms, characterized by the presence of motor asymmetry with bradykinesia (slowness), rigidity, resting tremor, and gait difficulties with postural instability [5], it is also accompanied by other non-motor symptoms such as cognitive impairment, behavioral disturbances, sleep disorders, hyposmia or autonomic dysfunction, among others [6,7]. As a result, PD is a highly heterogeneous disease, both with respect to its symptoms and its progression over time [8].

There is no cure for PD, but pharmacological and non-pharmacological treatments are available, providing symptomatic relief and improving the quality of life. In that regard, levodopa is the most effective medication available for treating the motor symptoms of PD, but in certain instances, it can be associated with other dopaminergic and non-dopaminergic drugs [9]. Other non-pharmacological treatments include deep brain stimulation of the subthalamic nucleus or the internal globus pallidum and physical, occupational, and neuropsychological interventions.

Early diagnosis of PD has profound implications for patients and their families, and despite important advances, it is still a challenge [10]. Recent developments include the validation of modified clinical diagnostic criteria, the introduction and testing of research criteria for prodromal Parkinson’s disease, and the identification of genetic subtypes and a growing number of biological biomarkers associated with Parkinson’s disease risk [10]. In this regard, the International PD and Movement Disorder Society (MDS) has published clinical criteria for the diagnosis of PD that are intended for use in clinical research and clinical practice. These criteria include two levels of certainty: clinically established PD (maximizing specificity, but with reduced sensitivity) and probable PD (balancing sensitivity and specificity) [11].

At present, the new MDS-revised version of the Unified Parkinson’s Disease Rating Scale (MDS-UPDRS) is used to rate the progress of PD. It comprises four parts: the non-motor aspects of daily life experiences (Part I); the motor aspects of daily life experiences (Part II); the motor examination (Part III); and, finally, the motor complications (Part IV) [12]. However, administering the full scale is very time-consuming, so in order to optimize time in clinical practice, several authors have tried to develop shorter rating scales or to use specific items from Part III of the MDS-UPDRS [13,14].

Among the motor features associated with PD, bradykinesia, characterized by hypokinesia (i.e., reduced movement amplitude, hesitations/halts, and sequence effect) has a significant impact on PD-related disability. Finger tapping (FT), one item of the motor examination included in the MDS-UPDRS, is a test in which the patient is asked to tap their index finger on their thumb as rapidly as possible, separating both fingers as much as possible. FT seems to be one of the most sensitive items, so it can be used to create a fast clinical judgment of motor status. Consequently, the FT test could potentially be used as the gold standard for video-based analysis [15]. However, interpreting objective bradykinesia data, obtained through kinematic techniques, is an especially challenging task, particularly when utilized for diagnostic purposes [16].

In recent years, there have been tremendous developments in the field of technologies which, coupled with the improved capabilities of machine learning (ML) algorithms, has led to increased research activity on the automatic monitoring of PD motor symptoms, the monitoring of the hands being of particular interest [17]. Recent publications have explored the use of AI in the diagnosis, progression, and assessments of PD motor and non-motor symptoms; however, there is limited research on the application of AI to video motion analyses. In this review, the aim is to highlight developing uses of AI-based technology for video motion analysis of hand movements, so as to facilitate the diagnosis, management, and empowerment of patients and to supervise the progression of their disease and their response to medication.

After this introduction, the remaining sections of this paper are as follows. Firstly, related works with automatic PD diagnosis are reviewed in Section 2. Secondly, all the papers on computer vision (CV) for PD diagnosis are carefully summarized in Section 3. Finally, the discussion of the results is presented in Section 4, and the main conclusions and future lines of research in Section 5 and Section 6 respectively.

## 2. Related Works

Over the past decade, researchers have been trying to define a useful and efficient method for PD assessment. This assessment could include diagnostic or/and PD ratings in accordance with UPDRS levels. As previously noted, the focus of this paper is on publications that use CV and FT; nevertheless, in the current section, some other approaches are covered in a brief review.

One of the initial attempts at diagnosing PD using CV includes the handwritten traces analysis [18]. In that test, patients have to fill out spirals and meanders on a piece of paper. Subsequently, the template and the drawings are identified and automatically split using image processing techniques; both were compared for feature extraction. Finally, for PD diagnosis (i.e., binary classification), they used traditional classifiers: Naïve Bayes, Optimum-Path Forest, and the Support Vector Machine (SVM) algorithm with a Radial Basis Function.

Historically, another common approach for PD assessment has been the use of external wearables [19] and sensors [20]. These devices are capable of recording movement-related data in effective and accurate ways, yet they are rarely used and are very expensive. It is a very interesting line of research, due to the reliability of the data capture methods that can extract solid features on the basis of a patient’s movements. In both cases [19,20], traditional classifiers, such as SVMs, *k*-Nearest Neighbors, and Decision Trees were used for PD diagnostis (i.e., classification). Jeon et al. [19] researched the use of a wristwatch-type wearable device with an accelerometer and a gyroscope to capture PD patients’ movement (acceleration, angular velocity, displacement, and angle) and, after the classification process, the authors performed a comparison of the UDPRS rating assigned by two neurologists. Related to Moshkova et al. [20], they focused their article on capturing hand movement signals from a LeapMotion sensor, which was placed at a distance of 15–30 cm from the patient’s hand. Their main aim was to perform PD assessment based on three different hand movements: FT, pronation–supination of the hand, and opening–closing hand movements.

In some studies [21,22,23], PD severity has been assessed through videos, thanks to the technological advances with CV systems. For example, Zhang et al. [21] focused on tremors, while Lu et al. [22,23] sought to quantify the severity of PD through the analysis of videos showing patients performing MDS-UPDRS. Zhang et al. [21] focused their research in analyzing tremor severity. For this purpose, they used OpenPose [24] for extracting 2D skeleton features; after this step, classification was performed using a graph neural network with a spatial attention mechanism. They also compared the results using other standard classifiers such as decision tree, convolutional neural network, and SVM. Meanwhile, Lu et al. [22,23] developed their own classifier, called Ordinal Focal Double-Features Double-Motion Network. In the previous paper [22], they only included gait analysis, but in the second one [23], FT was also evaluated. For gait analysis, they used VIBE [25] (video inference for human body pose and shape estimation) from extracting the 3D skeleton; for FT, they used the OpenPose [24] detection system.

A more recent article [17] must also be highlighted, which demonstrated the excellent correlation between data extracted using CV (more specifically MediaPipe [26]) and data captured with hand-held accelerometers. That study showed that non-intrusive methods such as CV can extract similar data to physical devices. Williams et al. [27] sought to prove a correlative relationship when assessing whether or not smartphone video recordings could be enough for evaluating bradikynesia. Standard smartphone video recordings of patients performing FT were tracked with DeepLabCut [28]. Three features such as tapping speed, amplitude, and rhythm were correlated with clinical ratings made by 22 movement disorder neurologists using the Modified Bradykinesia Rating Scale (MBRS) and the Movement Disorder Society revision of the MDS-UPDRS.

With a similar purpose in mind, Jaber et al. [29] sought to show how CV can be a suitable framework for PD assessment. The research showed a way of capturing FT movements using CV libraries (i.e., LabelImg and YOLO [30]) and transforming them into valuable metrics and features that can help with PD diagnostics.

Additionally, with the rise and democratization of Information Technology (IT), other researchers have approached PD assessment on the basis of IT interactions; for example, using mobile devices [31,32] and web browsers [33]. In both approaches, PD patients are invited to enter a gamified situation and are prompted to perform movements, either by tapping directly on the mobile phone screen [31,32] or by tracking mouse operations and keyboard inputs on the web browser [33]. Based on rhythm, accuracy, fatigue, and reaction time, among other factors, data are gathered for use as input for machine learning classifiers performing an assessment of PD. Regarding mobile phone usage, some of these investigations also took patient voice recordings into consideration [32].

Finally, the use of a CV framework for the diagnosis of movement disorders [34] or specifically PD [35] must be mentioned. In both papers, the way that deep-learning-based markerless motion tracking techniques can improve PD diagnosis and assessment were highlighted. Tien et al. [34] reviewed and discussed the potential clinical applications and technical limitations of these techniques, with a focus on DeepLabCut [28]. To evaluate the use of DeepLabCut for automated movement disorder disease assessment, they built a mobile frame with three synchronized cameras for recording hand movements, including healthy control subjects and movement disorder patients with various diagnoses, such as PD and essential tremor. In this case, authors share the utility of DeepLabCut, mentioning three ongoing studies, but without providing additional details. On the other hand, Sibley et al. [35] published an article wherein they reviewed the techniques, software libraries, and commercial approaches to video analyses of PD. Additionally, they identified challenges and possible solutions associated with rating motor symptoms of PD using video.

As it has been previously noted, traditional classifiers have been evaluated historically for appraising models’ performance and for carrying out statistical comparison with previous articles. To provide a summary view, Table 1 shows the classifiers which have been used in the articles mentioned in this section. After a first insight, decision tree, random forest, and SVM (in its different variations) seem to be the most used classifiers across the dissected articles. The use of ad hoc algorithms by some authors is also worth noting.

## 3. Finger Tapping and Computer Vision on Parkinson’s Disease Evaluation

In recent years, suitable methods have been proposed in several works for improving the diagnosis and/or the PD rating using CV and FT. In this section, the most interesting studies on CV and FT are briefly reviewed and compared. To do so, a state-of-the-art review is conducted with papers published no earlier than 2014 (e.g., [37]). As shown in Table 2, most of the papers were written over the past 4 years. There are two main reasons for this: first of all, the improvement in CV-related devices (cameras, smartphones, etc.) offers useful capabilities at affordable costs; secondly, the increasingly effective performance and accuracy of detection and pose estimation libraries [24,26,38] also make them effective choices.

**Figure 1:**
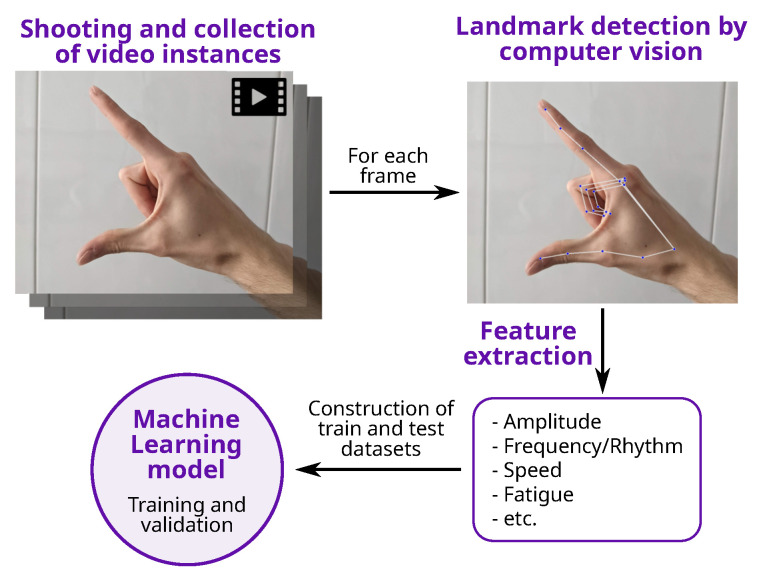
General process for the detection of PD using CV and FT.

All the studies share common points (see Figure 1): first of all, the thumb and the index fingers are automatically identified to perform feature extraction, before a dataset is compiled. Then, one or more algorithms are trained to produce a model, which can finally be used for testing with unseen instances/examples.

**Figure 2:**
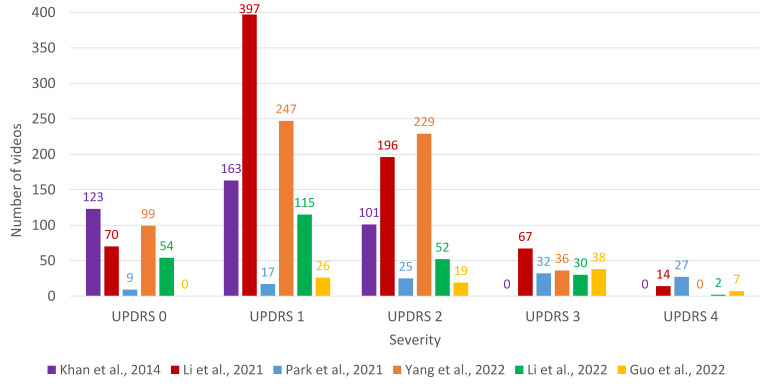
Barplot with the number of videos for each class (severity of UPDRS) for the papers that perform multiclass classification. References to the papers presented in the figure: Khan et al. [37], Li et al. [41], Park et al. [43], Yang et al. [44], Li et al. [45], and Guo et al. [46].

Most of the works are intended to solve classification problems: a few of them face the simplest problem (binary classification, i.e., either has PD or no PD), whereas most of them are designed to predict a class among more than two values (multiclass classification); for that purpose, the common approach is to predict the UPDRS rating. The distribution between classes is uneven (both for binary and multiclass classification papers), and a summary of the number of videos for each class is shown in Figure 2. It must be noted that UPDRS 3 and 4 level severity are especially underrepresented in most of the works.

The main characteristics of the research papers under analysis are summarized in Table 2.

### 3.1. Feature Extraction

As might be expected, the preference for deep neural networks for CV [48] meant that they were used for finger identification in all the studies. The most popular ones are listed below:

- Mediapipe [26] is an open-source framework, developed by Google, which provides real-time processing of multimedia data, including video and audio. It includes several modules for CV tasks, including pose estimation, face detection, hand detection, and object tracking.
- Openpose [24], developed by the Computer Vision Center at the Autonomous University of Barcelona, was released in 2016. It is a real-time multi-person human pose detection library with the capability of jointly detecting the human body, foot, hand, and facial keypoints on single images.
- MMPose [38] is an open-source toolbox for pose estimation based on PyTorch. It supports: multi-person human pose estimation, 133 keypoint whole-body human pose estimation, hand pose estimation, and 3D human mesh recovery.

The increasing use of Mediapipe must be noted in some of the most recent studies that were reviewed [17,45,47], replacing OpenPose [24], which was the most widely used in previous years [41,42,43].

The most common features used in the research papers are summarized in Table 3. One of the studies [41] was intentionally excluded from the table, due to a lack of information on the topic. Moreover, features used in no more than one paper were not included. At this point, it is important to mention the linguistic discrepancies between the notations in the different works, as well as the agreements that were reached for the purposes of this study on correct specification of the features that appear in the table.

- Amplitude and Speed: the two most common features to be analyzed for PD assessment using FT. Nevertheless, they are not considered in quite the same way in all works, although there are no semantic differences regarding the way that those features are to be understood in FT:
  – Amplitude: distance between thumb and index fingers.
  – Speed: amplitude difference over time.
  For example, a common approach is to obtain the values during the time series, but other authors also compute the mean or maximum value, a maximum value during the opening or the closing phases, minimum, standard deviation, etc. In other words, once the feature is considered, several metrics could be extracted, which will obviously differ across the different studies.
- Fatigue: this feature is evaluated in few articles, yet the approaches used for its estimation vary. It should be noted that it is not a physical value, such as amplitude and speed. The concept itself is similar in the different articles, but essential nuances in its estimation were identified. For example:
  – Difference between the highest and the lowest values of amplitude peaks [42].
  – Gradient in amplitude according to time [43].
  – Other authors [37] evaluated fatigue on the basis of different measures:
    * Difference between number of taps in two time slots.
    * Variation coefficient (VC) in tapping speed.
    * Difference between the average/VCs maximum amplitude of finger taps in two time slots.
    * VC in the maximum amplitude of finger taps.
    * Tapping acceleration.
- Frequency/Rhythm: without a doubt, the most abstract feature. Both concepts are used indistinctly, but not always for representing the same concept:
  – In some studies [17,27], its calculation is based on undertaking Fast Fourier Transform.
  – Another common approach [37,39] is to use a feature called “cross-correlation between the normalized peaks” (CCNP) for estimating consistency and rhythm in tapping.
  – Buongiorno et al. [40] used the averaged value of the division between the amplitude peak reached in a single exercise trial and the time duration of the trial.

### 3.2. Classifiers

As previously mentioned, all the papers that were reviewed had the common aim of performing a classification prediction (binary or multiclass). In other words, the main target of the studies was to implement a PD prediction according to UPDRS ratings for FT. In no more than a couple of studies [37,44] could the five levels of UPDRS not be predicted, due to the lack of enough examples during the training phase.

Different classifiers can be trained to perform the classification, once feature extraction has been completed, with SVM [49] (with its different variants/kernels) being by far the most popular [37,39,40,43,46,47].

Broadly used, especially in recent years, deep neural networks can also be applied as classifiers [40,44,45] (not only for finger identification) with different configurations and variations. Ad hoc designs and deployments have even been proposed [41].

On the other hand, multi-classifiers [50] (a.k.a. ensembles) are popular, the most widely used being random forest (RF) [42,46,47,51] and XGBoost [46,47,52]. Last but not least, conventional classifiers (such as Naïve Bayes [39,42], *k*-nearest neighbors [46,47], and logistic regression [39,42]) have commonly been used as baselines.

No insight could be given into which classifier was the best for the task of FT classification without further experimentation, due to the differences between the experimental setups, the datasets, the classification tasks, and the classifiers that were used.

### 3.3. Datasets

One of the main problems that severely complicates comparisons of the proposals is the lack of benchmark datasets. A particular dataset is used in every single study, usually containing small numbers of individuals: the smallest included 11 while the largest had 300. It must be noted that it can be extremely difficult to assess the performance of the proposals with such small-sized samples.

Deep analysis of this topic reveals great variability. Commonly, most of the research papers include PD patients and healthy controls (HCs). The problem here is that, sometimes, there are large differences related to class proportions. It is well known that imbalanced datasets (i.e., when a class is under-represented) are challenging, as algorithms will invariably ignore the underrepresented class/es. The class proportion is usually measured by means of the imbalance ratio (IR) [53].

$$IR=\frac{Number\;of\;majority}{Number\;of\;minority}$$

In this way, datasets can be further classified:

- Fairly balanced datasets (IR<2): the proportions between Parkinson’s disease patients and healthy controls are balanced [27,39,40,42,47].
- Imbalanced datasets (IR>2): the proportions are sufficient to take into account the imbalance problem [37,45,46].

In some studies, only videos from PD patients were taken into consideration [17,41,43,44]. There was no attempt to distinguish between PD patients and healthy controls, but only the differences between patients were considered, e.g., to rate them according to UPDRS.

### 3.4. Measures

After analyzing the articles, similar performance measures based on ML tasks were used. Brief explanations appear below alongside notes on their use in the papers that were reviewed.

- Accuracy: by far the most common measure [37,39,40,41,42,45,46,47], the foundation and common understanding of its meaning is what makes accuracy so popular (e.g., the number of successful outcomes divided by the total number of examples). Even a non-familiar reader could determine the achievement level by interpreting the accuracy percentage. However, this measure also has some drawbacks; a common complaint about accuracy is that it fails when the classes are imbalanced.
- Area Under ROC Curve (AUC): also considered a popular measure for classification problems [39,41,42]. It is commonly used in ML and data analytics to assess the performance of models at predicting binary outcomes and, in contrast to standard accuracy, it is particularly useful when processing imbalanced datasets, where accurate prediction of minority classes is of high importance.
- $F_{1}$-score [41,44,45,47] is the harmonic mean of precision (the number of true positive divided by the predicted as positives) and recall (the number of true positives divided by the number of all samples of the class of interest). $F_{1}$-score gives the same importance to both precision and recall, what can be considered as its main drawback [54]. Nonetheless, in real-world problems usually different costs are associated to different errors.

## 4. Discussion

First of all, as previously stated, PD assessment is still a major challenge for clinical neurologists. Few of the proposed solutions have been certified by the Movement Disorder Society for UPDRS rating. Despite the fact that this certification is meant to provide a common framework and to objectify a complex scenario, some voices are skeptical of its generalized use [55]. Firstly, previous knowledge is usually necessary as well as a case study with some clues on patient evolution, family background, and so on [39,42]. Secondly, some studies have shown some moderate agreement between neurologists and the rating provided for the same patients [46,55]. And finally, it is obvious that a subjective component is always present when the rating is provided by a human and it therefore depends to some extent on professional experience, among other things. On the other hand, the inclusion of several promising biomarkers including digital measures in future iterations for the clinic biomarkers PD staging system has been proposed [56]. In this regard, stages that distinguish between increasing degrees of motor impairment will be required to provide the space within which to develop sensitive quantitative measures including digital biomarkers [57].

Some authors have resorted to external devices in order to provide tools for improving the assessment of symptom severity among PD patients. Some can be mentioned here, such as wristwatch-type wearable devices [19], LeapMotion sensors [20], including depth cameras [46], motion controllers, i.e., Microsoft Kinect [40,47], and even trackers with reflective materials [40]. These external devices have some drawbacks. First, they are costly and specialized, which deter their generalized usage as effective devices. Second, they can alter the normal patient behavior, causing unusual movements.

Taking into account the previous arguments, CV appears to be the right technique for providing an objective and non-intrusive tool to perform PD assessment. The first articles which used CV for PD evaluation [37] needed a manual and/or additional calibration; nevertheless, the impressive technological improvements over recent years have led to the creation of self-provisioned models. Mobile phone cameras and webcams have increased their performance exponentially, being able to capture images and videos at very high resolution and recording them at different frequencies (frames per second). Furthermore, several software libraries and frameworks that have high accuracy rates have been developed for object and human detection. Some of those libraries, previously used for PD assessment and diagnosis, have yielded promising results: OpenPose [41,42], DeepLabCut [27,34], MediaPipe [17,45,47], etc. It must be noted that most of the studies used 2D capture, while 3D capture was only selected in a few of the studies [40,46,47]. In strictly technical terms, 3D capture systems can perform FT detection better, but 2D capture is arguably still preferable for several reasons. The 3D specialized systems are more expensive and complex to install and use. Other 3D capture systems like Microsoft Kinect are more accessible, but they still need a controlled environment and achieve similar results to 2D capture using a general purpose camera. The need to install specialized systems that require a controlled environment and, in some cases, specialized personnel, can result in delays in accessing PD assessment and/or diagnostics, which defeats the purpose of such systems. Two-dimensional cameras, on the other hand, are already in most doctor’s offices and do not require any special training. At this point in time, the performance of basic and inexpensive vision-related devices continues to improve, offering exciting new possibilities.

Combining these two improvements and the use of AI and ML techniques, all the tools are in place for precise assessments of human movements. Once movement has been accurately detected and converted into time series data, machine learning, which has shown its efficiency for PD assessments, is the next step; for example, SVM [20,37,39,40,43,46,58], extreme gradient boosting [46,47], *k*-nearest neighbors [46,47], random forest [42,46,47], and Naive Bayes [39,42].

Regarding the classifiers used, they can usually be grouped into white and black boxes, according to its interpretability [59]. Black-box classifiers, such as SVMs, are able to achieve high accuracy but it is difficult (if not impossible) to explain why. On the other hand, white-box classifiers, such as decision trees, can be easily interpretable for a human. In some contexts, as the medical one, explainability is essential. Experts need to know why a decision is taken: which features are involved, in which ranges... Interpretable models are preferred in some occasions even when they are not the best ones for the task at hand.

Nonetheless, the interpretation of the data detecting FT bradykinesia using AI and ML techniques should be taken with caution. Information about the association of motor impairment detected by these techniques with functionality, and impairment of daily living activities should be taken into consideration. In addition, by using the FT impairment information exclusively, we are losing information about other disabling motor signs of PD such as gait impairment.

## 5. Conclusions

As previously noted, PD assessment is a great challenge in itself and the use of non-invasive techniques is the accepted path toward improved assessment. On the one hand, it makes it possible to take advantage of CV performance improvement and its democratization, a generalized use without incurring high expenses. On the other hand, ML algorithms have already shown themselves to be an efficient movement disorder classification methodology.

Following the review of relevant papers, some insights can be provided into the objective characterization of FT using CV technologies. FT is one motor task, among many others that are commonly assessed in clinical neurology, due to its ease of performance and evaluation, after which the severity of the symptoms of bradikynesia may be diagnosed and related to functional impairment. Advances in CV offer promising results that can assist physicians when assessing PD. However, the differences between the studies have made it impossible to compare the performance of the various proposals.

With this in mind, some recommendations must be taken into account for the future of automatic FT identification. One of the main concerns is the lack of public benchmarks of FT videos, which means that the studies cannot be reproduced and compared. The proportional distribution of the classes must also be considered with regard to the datasets, because the imbalance ratio on some of them can mean some measures such as accuracy are misleading. Thus, a proper classifier (i.e., one that can address imbalanced problems) and a proper measure (i.e., one that is insensitive to imbalance) are mandatory for future studies. Another aspect concerns the irregular usage of feature names in the different studies, which make it extremely difficult to ascertain the exact features that were used. Finally, the FT bradykinesia information using AI and ML techniques should be interpreted in the context of related functional impairment.

## 6. Future Work

In accordance with the majority of studies included in this review, it is necessary to conduct studies with a greater number of FT videos at different stages of PD and to classify FT according to functional impairment. In doing so, an ordered classification based on the MDS-UPDRS scale could be achieved. Furthermore, it will be essential to expand the scope of this type of studies, not only for the evaluation of bradykinesia in PD, but also for other motor disorders that can be characterized through CV.

As previously noted, the studies under review were focused on classification and not on the level of impairment. In this regard, in terms of impairment, we should consider that much of the data captured by FT videos could be underrepresented or not well assessed by the MDS-UPDRS. On the contrary, digital quantification of FT and related impairment could be interpreted differently by the neurologist using the MDS-UPDRS. Therefore, there is an inherent order between the different classes, and ordinal classification or ordinal regression may be more advisable [60,61]. Further studies designed to study FT prediction for PD using ordinal classification should be encouraged.

## Figures and Tables

**Table 1:** Summary of the related works and the classifiers used in each study. When more than one classifier was used, the one that achieved the best performance is highlighted in bold (SVM: support vector machine).

Ref.	Year	Brief Description	Classifiers
[18]	2016	Handwritten traces	Naïve Bayes Optimum-Path Forest **SVM-R**
[19]	2017	Tremor severity analysis using a wristwatch-type wearable device	**Decision tree** SVM-L SVM-P SVM-R *k*-nearest neighbors Discriminant Analysis
[31]	2018	On-screen tapping on a mobile phone (iPhone app)	Logistic regression Random forest Deep Neural Network **Convolutional Neural Network**
[32]	2018	PD-related activities (voice, finger tapping, gait, balance, and reaction time) assessment (Android app)	Machine-Learning algorithm [36]
[20]	2019	Hand movements data capturing using LeapMotion sensor	Decision tree **SVM** *k*-nearest neighbors Random forest
[22]	2020	Gait analysis using videos	Ordinal Focal Double-Features Double-Motion Network
[23]	2021	Gait and FT analysis using videos	Ordinal Focal Double-Features Double-Motion Network
[21]	2022	Tremor severity analysis using videos	**Graph Neural Network** Decision tree Convolutional Neural Network SVM
[33]	2023	Gamified website tracking keyboard and mouse inputs	**Random forest** Decision tree SVM Multilayer perceptron

**Table 2:** Summary of the main characteristics of the computer vision (CV)-related papers reviewed in this research. In the studies with more than one classifier, the one that achieved the best performance is highlighted in bold (PD: Parkinson’s disease, HC: healthy control, AUC: area under the ROC, SVM: support vector machine).

Ref.	Year	Capture	Finger Identification	Classifiers	ML Problem	Dataset	Performance Measures
[37]	2014	2D	OpenCV	SVM-PUK	Binary Multiclass (3)	13 PD 6 HC	Accuracy
[39]	2019	2D	Convolutional Neural Network	**Naïve Bayes** **Logistic regression** SVM-L SVM-R	Binary	20 PD 15 HC	Accuracy Sensitivity Specificity AUC
[40]	2019	3D	Custom-made trackers	Artificial neural network **SVM**	Binary	16 PD 14 HC	Accuracy Sensitivity Specificity
[41]	2021	2D	OpenPose	Convolutional Neural Network	Multiclass (5)	157 PD 0 HC	Accuracy AUC Precision Recall $F_{1}$-score
[42]	2021	2D	Single Shot MultiBox Detector (SSD) + OpenPose	**Logistic Regression** Naïve Bayes Random forest	Binary	22 PD 20 HC	Accuracy AUC
[43]	2021	2D	OpenPose	SVM-R	Multiclass (5)	55 PD 0 HC	Weighted κ Intraclass corr. coeff.
[44]	2022	2D	MMPose	Deep Neural Network	Multiclass (4)	300 PD 0 HC	Precision Recall $F_{1}$-score
[45]	2022	2D	MediaPipe	Fully Connected Network	Multiclass (5)	93 PD 27 HC	Accuracy Precision Recall $F_{1}$-score
[46]	2022	3D	Spatial-Temporal Anchor-to-Joint Regression Network (ST-A2J)	*k*-nearest neighbors Random forest XGBoost SVM-L **SVM-R**	Multiclass (5)	48 PD 11 HC	Accuracy
[47]	2023	3D	MediaPipe	**k-nearest neighbors** Random Forest XGBoost SVM	Binary	35 PD 60 HC	Accuracy Precision Recall $F_{1}$-score

**Table 3:** Summary of finger tapping features used in the CV papers reviewed in this research. The symbol () represents that the feature is used in the work.

Feature	[37]	[39]	[40]	[27]	[42]	[43]	[29]	[44]	[45]	[46]	[17]	[47]
Amplitude	✓	✓	✓	✓	✓	✓	✓	✓	✓	✓	✓	✓
Frequency/Rhythm	✓	✓	✓	✓			✓				✓	
Speed	✓		✓	✓	✓	✓	✓	✓	✓	✓		✓
Fatigue	✓				✓	✓						

## Data Availability

Data sharing is not applicable.
